# Human Cytomegalovirus Infection Dysregulates the Canonical Wnt/β-catenin Signaling Pathway

**DOI:** 10.1371/journal.ppat.1002959

**Published:** 2012-10-11

**Authors:** Magdalena Angelova, Kevin Zwezdaryk, MaryBeth Ferris, Bin Shan, Cindy A. Morris, Deborah E. Sullivan

**Affiliations:** 1 Department of Microbiology and Immunology, Tulane University School of Medicine, New Orleans, Louisiana, United States of America; 2 Department of Medicine, Section of Pulmonary Diseases, Critical Care and Environmental Medicine, Tulane University School of Medicine, New Orleans, Louisiana, United States of America; University of Alabama at Birmingham, United States of America

## Abstract

Human Cytomegalovirus (HCMV) is a ubiquitous herpesvirus that currently infects a large percentage of the world population. Although usually asymptomatic in healthy individuals, HCMV infection during pregnancy may cause spontaneous abortions, premature delivery, or permanent neurological disabilities in infants infected *in utero*. During infection, the virus exerts control over a multitude of host signaling pathways. Wnt/β-catenin signaling, an essential pathway involved in cell cycle control, differentiation, embryonic development, placentation and metastasis, is frequently dysregulated by viruses. How HCMV infection affects this critical pathway is not currently known. In this study, we demonstrate that HCMV dysregulates Wnt/β-catenin signaling in dermal fibroblasts and human placental extravillous trophoblasts. Infection inhibits Wnt-induced transcriptional activity of β-catenin and expression of β-catenin target genes in these cells. HCMV infection leads to β-catenin protein accumulation in a discrete juxtanuclear region. Levels of β-catenin in membrane-associated and cytosolic pools, as well as nuclear β-catenin, are reduced after infection; while transcription of the *β-catenin* gene is unchanged, suggesting enhanced degradation. Given the critical role of Wnt/β-catenin signaling in cellular processes, these findings represent a novel and important mechanism whereby HCMV disrupts normal cellular function.

## Author Summary

A large percentage of the world population is infected with HCMV. As a leading viral cause of birth defects in the developed world, HCMV represents a significant public health burden. For the first time, we report that HCMV infection dysregulates the canonical Wnt/β-catenin signaling pathway which is essential in regulating a diverse range of biological functions. We demonstrate that HCMV infection leads to sequestration and degradation of β-catenin protein, the effector transcription factor in the pathway, thus preventing its downstream signaling activities. Since this pathway is essential in regulating mammalian development and homeostasis, the finding that HCMV impairs this pathway becomes globally important for understanding viral pathogenesis, particularly that related to HCMV disease.

## Introduction

Human Cytomegalovirus (HCMV) is a betaherpesvirus that is ubiquitously present in the human population. Infection with HCMV is usually subclinical in healthy adults, but can cause serious disease in populations with underdeveloped or compromised immune systems [1]. HCMV is the leading viral cause of congenital birth defects in the developed world [2], [3]. Although the majority of congenitally infected infants are asymptomatic at birth, 5 to 20 percent of the infected neonates are born with symptoms. About 5 percent of symptomatic children die during the neonatal period and the majority of those who survive develop permanent neurodevelopmental complications, such as hearing loss and mental retardation [4], [5]. A small percentage of infants who lack symptoms may also suffer long-term neurological abnormalities later in life [6]. Opportunistic infections with HCMV also increase morbidity in immunocompromised individuals, such as organ transplant recipients and AIDS patients [1]. In addition, infection with HCMV compromises the success of bone marrow and solid organ transplantations, where it is associated with graft rejection [7]. Recent evidence also implicates HCMV infection as a contributing factor in the development of atherosclerosis and cardiovascular disease [8].

Upon entry into the cell, HCMV manipulates the host environment in order to establish productive infection and ensure progression of the viral replication cycle. HCMV controls the host cell through dysregulating a multitude of signaling, cytoskeletal and regulatory pathways that control key cellular events. HCMV is known to highjack the host replication machinery [9], alter the cell cycle [10]–[12], and manipulate the host innate and adaptive immune responses (reviewed in [13]).

One important signaling pathway that has been reported to be perturbed by herpesviruses is the canonical Wnt/β-catenin pathway (reviewed in [14]). This signaling pathway is crucial for driving a large number of the molecular events that take place during embryonic development, such as cell fate determination and establishment of tissue polarity [15] as well as playing a critical role in differentiation of multiple cell types including neurons [16], [17] and mesenchymal stem cells [18]. Recent evidence also demonstrates that activation of this pathway is indispensible for differentiation of fetal cytotrophoblasts into an invasive phenotype during placentation [19]. Apart from its role in development, canonical Wnt signaling is involved in regulating many homeostatic events in adult tissues. Among the processes regulated by Wnt signaling are cell proliferation, motility, survival, and stem cell maintenance [20]–[22]. Aberrant activation of this pathway is associated with the onset of several types of human malignancies [23], [24].

β-catenin, the major effector protein in the canonical Wnt signaling pathway, is normally retained in the cytoplasm in an inactive state through its interaction with a large protein complex comprised of axin, adenomatous polyposis coli (APC) and two serine/threonine kinases: glycogen synthase kinase-3β (GSK-3β) and casein kinase 1 (CK1). This complex maintains low levels of β-catenin in the cell through constant proteasome-mediated degradation. Phosphorylation of serine-45 by CK1 and subsequent phosphorylation on serine-33 and -37 by GSK-3β marks β-catenin for polyubiquitination and proteolytic degradation. Pathway activation is initiated when Wnt ligands, a large family of secreted glycoproteins, bind to heterodimeric Frizzled (FZD)/low-density lipoprotein receptor-related protein-5/6 (LRP-5/6) receptors on the surface of target cells. This initiates a cascade of events leading to phosphorylation and activation of Disheveled (Dsh/Dvl), a cytoplasmic scaffolding protein that relays Wnt signaling downstream and disrupts the axin/APC/GSK-3β complex. Disruption of the complex causes accumulation of stable, hypophosphorylated β-catenin in the cytoplasm, followed by its translocation to the nucleus (reviewed in [25]). Once in the nucleus, β-catenin binds T cell-specific factor (TCF)/lymphoid enhancer-binding factor-1 (LEF-1) DNA-binding factors to activate transcription of over fifty target genes involved in cell maintenance, proliferation and survival, such as *cyclin D1*, *c-myc*, *metalloproteinase-2 (MMP-2)* and *metalloproteinase-9 (MMP-9)* [26]–[28].

To date, the effect of HCMV infection on this essential signaling pathway has not been reported. In this study, we demonstrate that HCMV infection induces juxtanuclear accumulation and degradation of β-catenin resulting in a decrease in its transcriptional activity in response to Wnt ligand stimulation. Diminished activation of this important pathway introduces a novel mechanism whereby HCMV causes impaired cellular function.

## Results

### HCMV infection inhibits Wnt/β-catenin transcriptional activity

**Figure 1:**
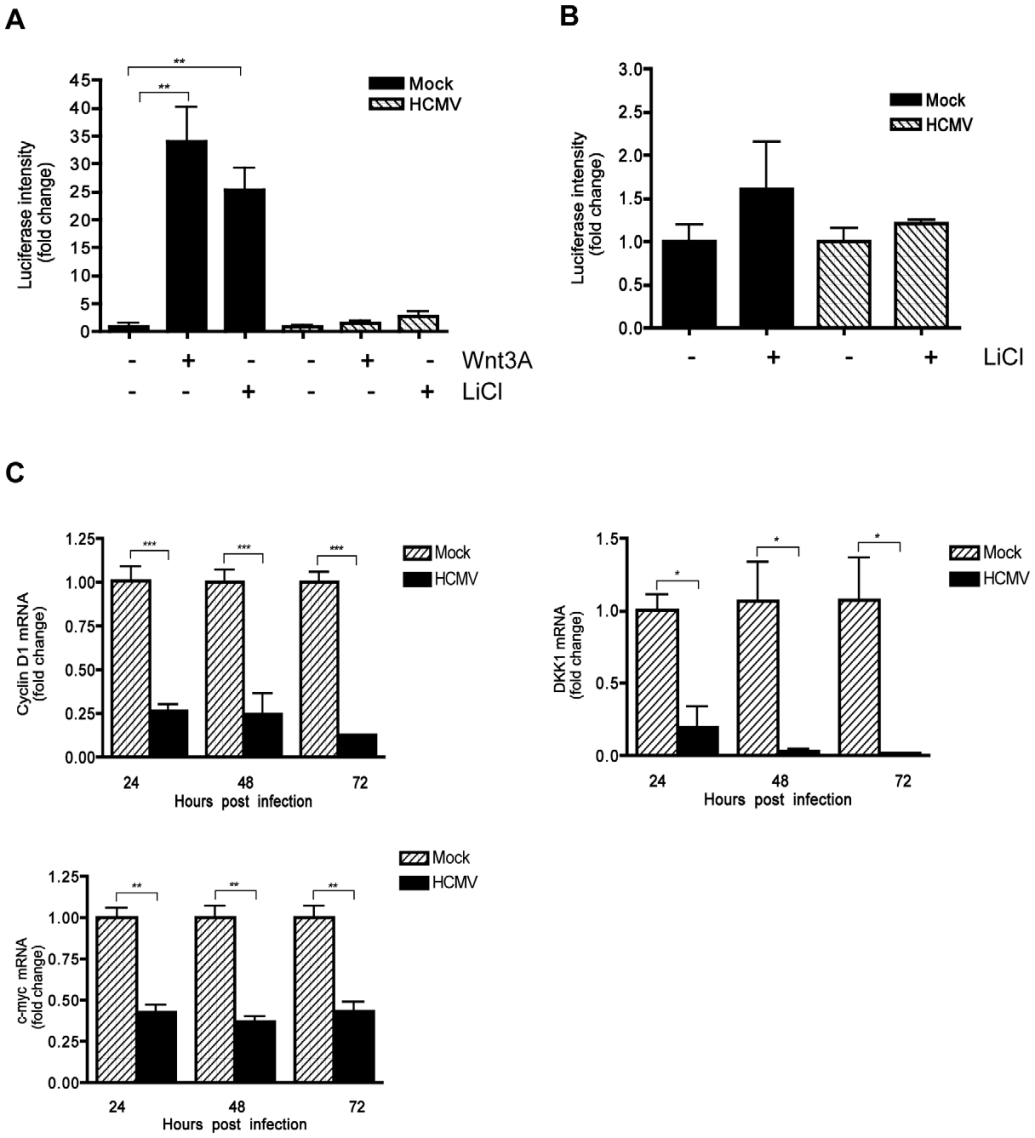
Inhibition of β-catenin transcriptional activity by HCMV. (A) HFFs were co-transfected with TOPflash and pRL-TK plasmids and 12 hr later infected with HCMV-TR (MOI of 1) or mock-infected. Forty eight hr after infection, the cells were stimulated with either 150 ng/mL Wnt-3A, 20 mM LiCl or PBS control for an additional 12 hr. Luciferase expression in cell lysates was measured and normalized to *Renilla* luciferase activity. Data are presented as the mean of results from two independent experiments each performed with triplicate transfections. **p<0.01. (B) HFFs were transfected with FOPflash plasmid (as negative control) and pRL-TK control plasmid and mock- or TR-infected, then stimulated with LiCl and analyzed as described in (A). (C) *Cyclin D1*, *DKK1* and *c-myc* mRNA levels in HFFs were analyzed from triplicate infections by qRT-PCR at 24, 48 and 72 hr after infection with HCMV-TR (MOI of 1). Each sample was analyzed in triplicate and normalized to the level of 36B4. The data are presented as fold change (mean +/− SEM) relative to the mock-infected sample at each corresponding time point. *p<0.05; **p<0.01; ***p<0.001.

The TCF/LEF-1-luciferase reporter construct TOPflash was used to determine whether HCMV infection affects β-catenin activity. Human foreskin fibroblasts (HFFs) were transiently transfected with TOPflash or FOPflash containing mutated TCF/LEF-1 binding sites as a control. After 12 hr, transfected cells were infected with HCMV or mock-infected. At 48 hr after infection, the cells were stimulated with the canonical ligand Wnt-3A or lithium chloride (LiCl) for an additional 12 hr and then analyzed for luciferase expression. LiCl is a potent activator of Wnt signaling that acts downstream of Wnt receptors by inhibiting GSK-3β thus stabilizing β-catenin and promoting nuclear translocation [29]. Stimulation of mock-infected cells with both Wnt-3A and LiCl markedly increased luciferase expression compared to the PBS-treated control (Figure 1A). In contrast, TOPflash activity in HCMV-infected cells stimulated with Wnt-3A or LiCl was only slightly increased from basal levels (Figure 1A), suggesting inhibited activation of the TCF/LEF-1 transcription complex. Inhibition of both Wnt-3A- and LiCl- stimulated TOPflash activity suggests that the inhibitory activity is at the level of β-catenin and not at the level of Wnt receptors. LiCl treatment of FOPflash transfected cells failed to induce luciferase expression in either mock- or HCMV-infected cells (Figure 1B), demonstrating the specificity of TOPflash activity.

Next, the expression of endogenous Wnt/β-catenin target genes was examined in HCMV-infected cells. Expression of *cyclin D1*, *c-myc* and *Dikkopf-1* (*DKK1*) genes, which are transcriptionally regulated by the β-catenin/TCF/LEF-1 complex [26], [27], [30], was evaluated in HCMV-infected HFFs by quantitative real-time RT-PCR (qRT-PCR) analyses. HCMV infection led to modest but significant downregulation of expression of all three genes, compared to mock-infected cells (Figure 1C). Together, these results demonstrate that HCMV inhibits the transcriptional activity of the β-catenin/TCF/LEF-1 complex.

### HCMV infection induces juxtanuclear accumulation of β-catenin

**Figure 2:**
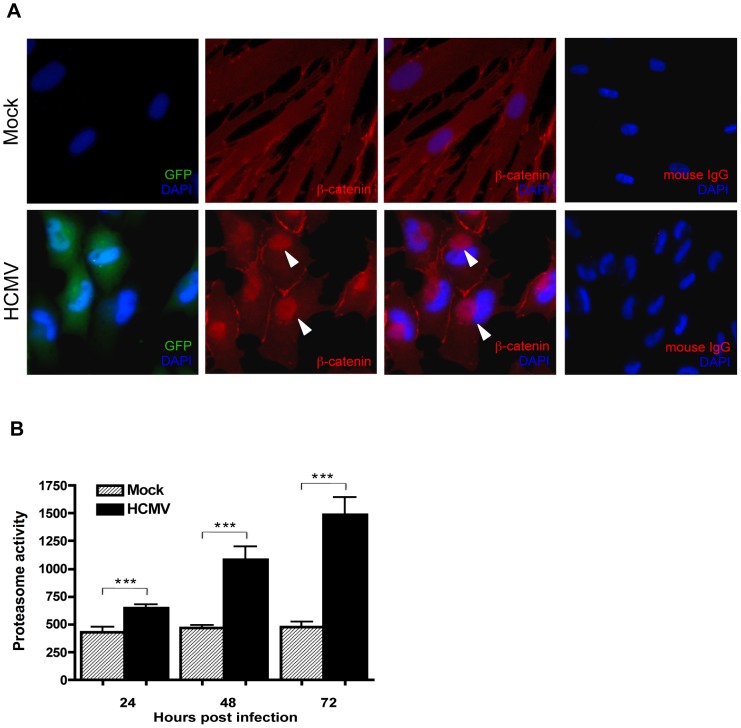
β-catenin aggregates at a central juxtanuclear location in HCMV-infected HFFs. (A) HFFs were seeded on glass coverslips and infected with Towne-GFP (MOI of 1–2) for 48 hr. Cells were stained with mouse monoclonal antibody to β-catenin followed by AlexaFluor 555-conjugated anti-mouse IgG. Nuclei were counterstained with DAPI. Normal mouse IgG was used as an isotype control. GFP positive cells represent infected cells. Arrowheads indicate aggregation of β-catenin in infected cells. (B) Cell extracts collected from four separate wells of HHFs at 24, 48 or 72 hr after infection with HCMV-TR (MOI of 1–2) were analyzed for the chymotrypsin-like activity of the 26S proteasome by addition of a fluorogenic peptide substrate (Suc-LLVY-AMC). Proteasome activity is expressed as relative fluorescence units and reported as the mean ± SEM (n = 4) and is representative of two independent experiments. ***p<0.001.

The effect of HCMV infection on the subcellular distribution of β-catenin in HCMV-infected HFFs was investigated by immunofluorescence. HFFs that had been infected with HCMV or mock-infected for 48 hr were immunostained for β-catenin (Figure 2A). In mock-infected cells, β-catenin displayed typical diffuse cytoplasmic/membranous staining. In contrast, intense β-catenin staining was observed in a distinct juxtanuclear region in HCMV-infected cells that was absent in mock-infected cells. The morphology and localization of the β-catenin aggregates observed in HCMV infected cells resembles that of aggresomes which typically form at the microtubule-organizing center (MTOC) in response to accumulation of misfolded proteins or when the capacity of the proteasome degradation machinery is inhibited or overwhelmed [31]. To determine if aggregation of β-catenin was a result of proteasomal impairment, protein lysates were collected from HFFs at 24, 48 and 72 hr after infection with HCMV and analyzed for catalytic activity of the 26S proteasome using a fluorophore-labeled proteasome specific substrate Suc-LLVY-AMC. Consistent with a previous report [32], there was a significant increase in the catalytic activity of the proteasome in HCMV-infected cells (Figure 2B). This suggests that HCMV-induced aggregation of β-catenin is not a result of impaired proteasome function that prevents the normal turnover of β-catenin.

### HCMV infection promotes degradation of β-catenin

**Figure 3:**
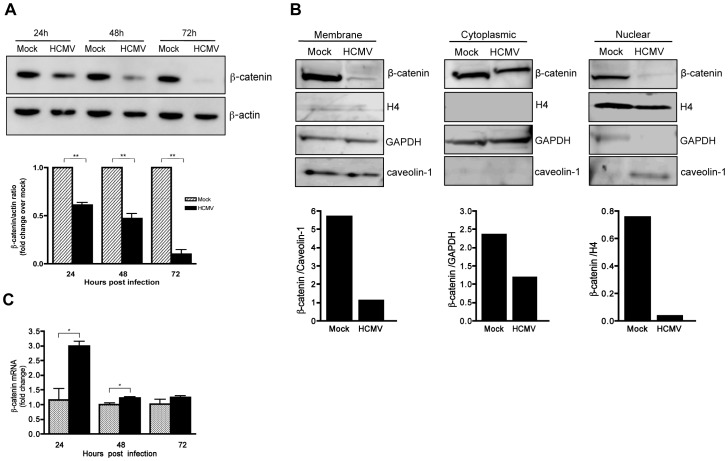
HCMV induces degradation of β-catenin. (A) Lysates were collected from HFFs infected with HCMV-TR (MOI of 1–2) at the indicated times post infection and analyzed for β-catenin expression by Western blot. β-actin served as a loading control. β-catenin protein levels were quantitated by densitometric analysis using ImageJ software (n = 3) and normalized to mock-infected cells at each time point, which was set to a value of 1.0. Data are presented as the mean ± SEM of 3 independent experiments. **p<0.01 (B) Membrane, cytoplasmic and nuclear protein-enriched cell fractions were prepared from HFFs 48 hr after infection with HCMV-TR (MOI of 1–2), and analyzed for β-catenin expression by Western blot. Caveolin-1, GAPDH, and histone H4 served as loading controls for the membrane, cytoplasmic and nuclear fractions, respectively. β-catenin protein levels were quantitated by densitometric analysis using ImageJ software. (C) β-catenin mRNA expression in HCMV-TR-infected (MOI of 1–2) or mock-infected HFFs at 24, 48 and 72 hr post infection were analyzed by qRT-PCR. Relative β-catenin mRNA levels in virus-infected samples at each timepoint were normalized to GAPDH mRNA levels and expressed as fold change relative to the corresponding uninfected control. Data are presented as mean +/− SEM of 3 independent experiments. *p<0.05.

To determine if HCMV infection alters β-catenin protein levels, HFFs were infected with HCMV and analyzed for total β-catenin by Western blotting at various times after infection. There was a significant (up to 90%) reduction in the total levels of β-catenin protein in HCMV-infected HFFs by 72 hr after infection (Figure 3A). Apart from being a key component of canonical Wnt signaling, β-catenin is also an integral part of adherens junctions where it mediates contact between cadherins and the actin cytoskeleton. Cytoplasmic, nuclear and adherens junctions-associatedβ-catenin constitute distinct cellular pools of β-catenin that are tightly regulated. To determine if HCMV infection differentially affects the levels of β-catenin between subcellular compartments, membrane, cytoplasmic and nuclear fractions were isolated from mock and HCMV-infected HFFs and analyzed for β-catenin by Western blotting. Histone H4, glyceraldehyde 3-phosphate dehydrogenase (GAPDH) and caveolin-1 were used as markers of the purity of the nuclear [33], cytoplasmic [34] and membrane cell fractions [35], respectively. HCMV infection reduced the amount of cytoplasmic, membrane and nuclear β-catenin compared to mock-infected controls. Although there was some cross contamination between fractions, the results suggest that levels of β-catenin in all three fractions were reduced in HCMV infected cells (Figure 3B).

β-catenin levels are normally regulated by phosphorylation-dependent degradation via the ubiquitin-proteasome system [36]. However, regulation of β-catenin at the level of transcription has been reported [37]. To determine if HCMV infection affects β-catenin gene transcription, qRT-PCR was performed. As indicated in Figure 3C, β-catenin mRNA levels were slightly higher in HCMV-infected HFFs compared to mock-infected controls, indicating that the decrease in β-catenin protein induced by HCMV is posttranscriptional.

### HCMV gene expression is required for HCMV-induced juxtanuclear accumulation and degradation of β-catenin

**Figure 4:**
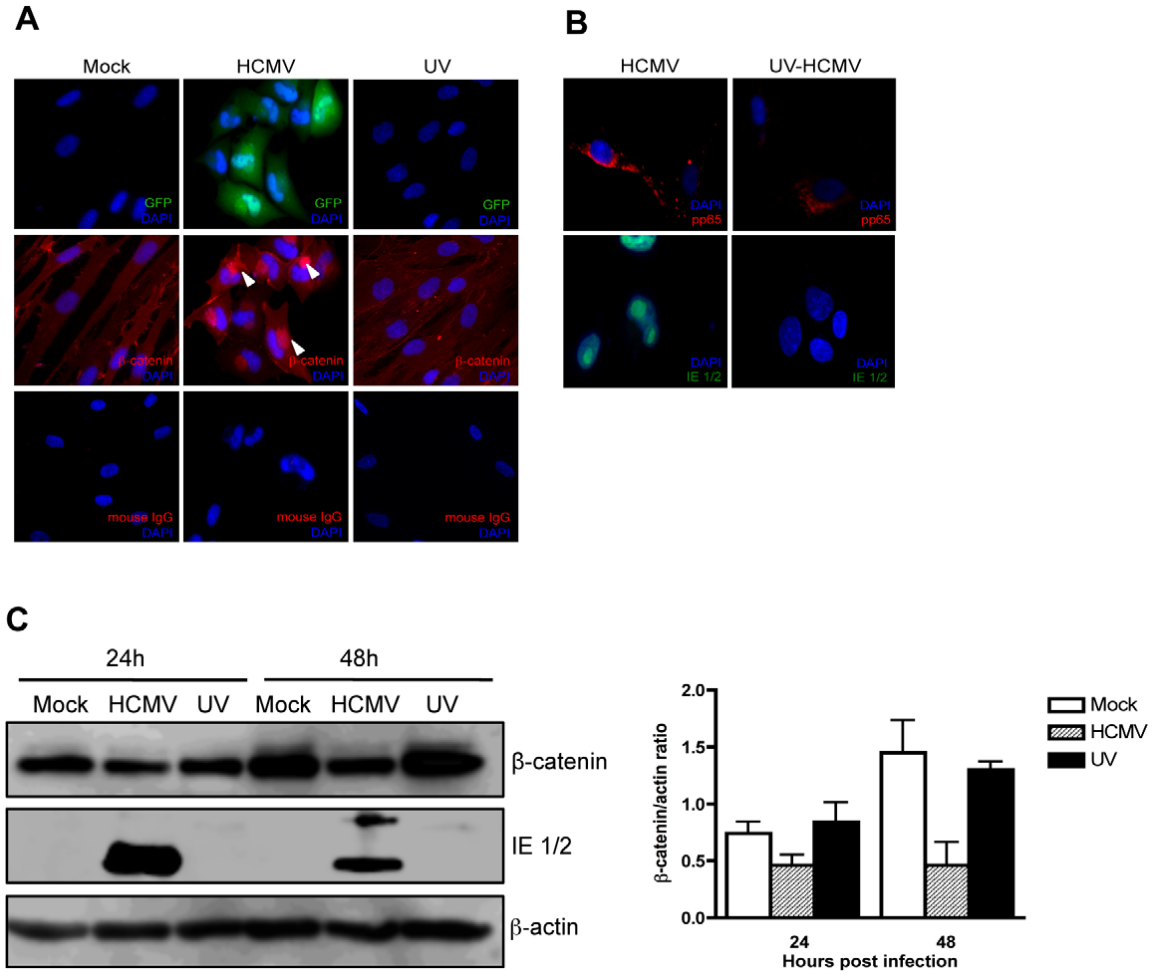
Effect of UV-inactivated HCMV on β-catenin degradation. (A) HFFs grown on chamber slides were infected with Towne-GFP (MOI of 1–2), UV-irradiated Towne-GFP or mock-infected and fixed at 48 hr post infection. The cells were stained with mouse anti-β-catenin antibody followed by AlexaFluor 555-conjugated anti-mouse IgG. Nuclei were counterstained with DAPI. Mouse IgG was used as an isotype control. GFP positive cells represent infected cells. Arrowheads indicate aggregation of β-catenin in infected cells. (B) Immunofuorescence detection of the viral tegument protein pp65 (*red*) and immediate early proteins (green) in HFFs infected (MOI of 1–2) with HCMV-TR and UV-irradiated HCMV-TR at 6 hr (pp65) and 24 hr (IE1/2) post infection. (C) HFFs were infected with HCMV-TR, UV-irradiated HCMV-TR or mock-infected (MOI of 1–2). Protein lysates were collected at 24 and 48 hr post infection and analyzed for β-catenin and HCMV IE 1/2 expression by Western blot followed by quantitative densitometric analysis using ImageJ software. β-actin served as a loading control. Data are presented as mean +/− SEM of 2 independent experiments.

To determine whether β-catenin degradation requires *de novo* viral gene expression, HFFs were infected with HCMV or ultraviolet (UV)-inactivated HCMV. Immunofluorescence staining 24 hr after infection detected juxtanuclear accumulation of β-catenin in cells infected with HCMV virus, whereas in cells infected with UV-inactivated virions β-catenin failed to aggregate (Figure 4A). Entry of UV-inactivated virions was confirmed by tegument-associated pp65 protein staining in infected cells 6 hr after infection (Figure 4B). Successful inactivation of the virus was confirmed by the absence of HCMV immediate early (IE)1/2 protein expression in infected cells (Figure 4C). In addition, β-catenin levels were analyzed by Western blotting in cells infected with HCMV or UV-inactivated virus, at 24 and 48 hr after infection. HCMV, but not UV-inactivated virus reduced protein levels of β-catenin at each time point (Figure 4C). This suggests that degradation of β-catenin is not due to HCMV binding and entry into the cell or to tegument-associated proteins, but rather requires viral gene expression.

### HCMV inhibits Wnt/β-catenin signaling in human extravillous cytotrophoblasts

**Figure 5:**
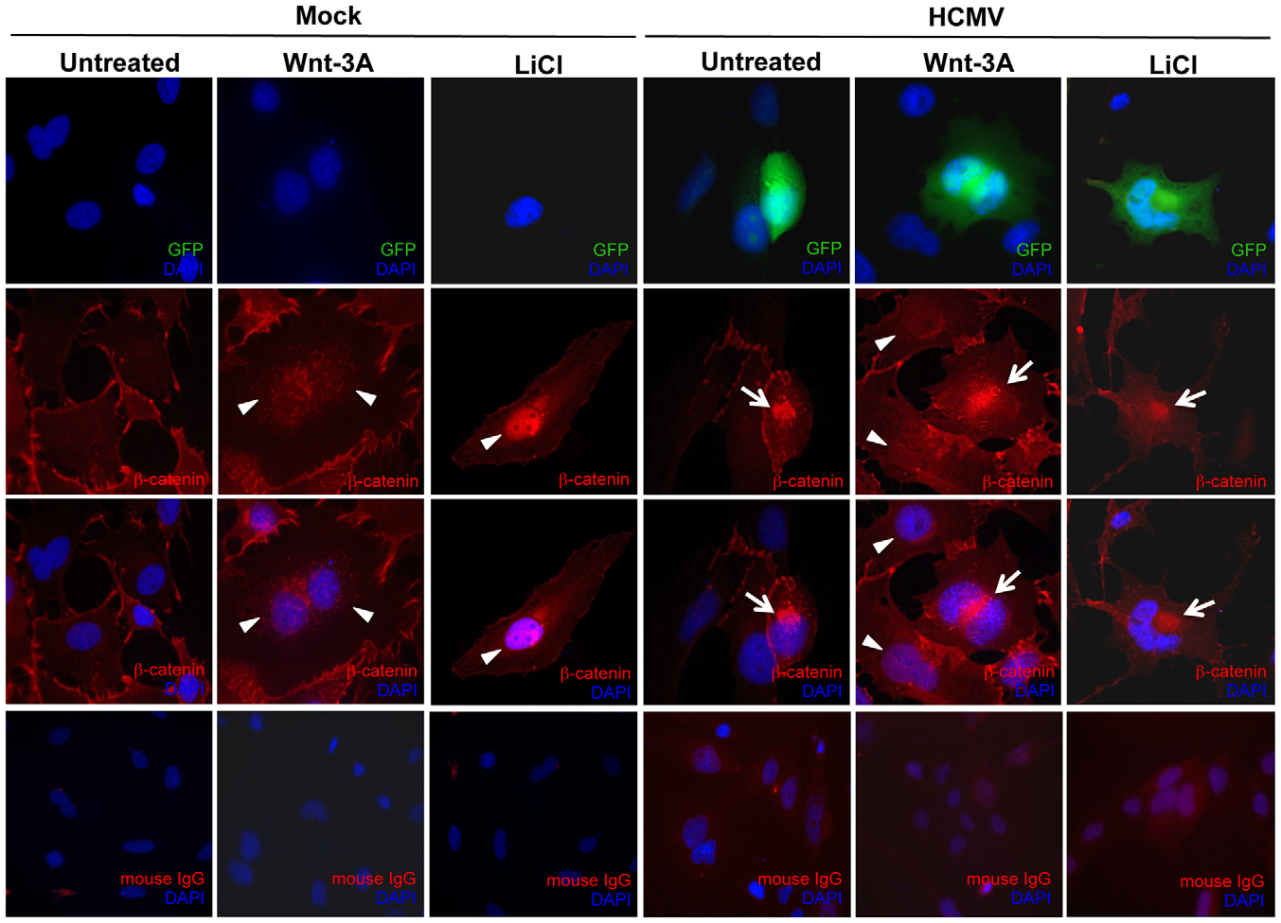
HCMV infection inhibits β-catenin nuclear translocation in EVTs. SGHPL-4 cells were seeded on glass coverslips and infected with Towne-GFP (MOI of 1–2) or mock-infected for 48 hr. Cells were then treated with 150 ng/ml Wnt-3A or 20 mM LiCl for 6 hr followed by immunostaining for β-catenin using a mouse anti-β catenin antibody, followed by a goat anti-mouse secondary IgG conjugated to AlexaFluor 555. Nuclei were counterstained with DAPI. Mouse IgG was used as an isotype control. GFP positive cells represent infected cells. Arrowheads point to nuclear accumulation of β-catenin. Arrows indicate aggregation of β-catenin in infected cells.

Wnt signaling plays a major role in placental development and morphogenesis. We have shown previously that HCMV inhibits differentiation of SGHPL-4 cells, an extravillous trophoblast (EVT) cell line derived from first trimester placenta, into a migratory and invasive phenotype [38]. To determine whether HCMV infection inhibits Wnt signaling in SGHPL-4 cells as was observed in HFFs, SGHPL-4 cells that had been infected with HCMV for 48 hr, were stimulated with Wnt-3A ligand or LiCl for 6 hr and then immunostained for β-catenin (Figure 5). Both Wnt-3A and LiCl treatments led to translocation of β-catenin to the nucleus of mock-infected cells. In contrast, β-catenin was seen to aggregate in distinct juxtanuclear region in HCMV-infected cells. Moreover, Wnt-3A- or LiCl-treatment of infected cells showed no change in β-catenin localization. Mock-infected cells showed no aggregation of β-catenin at any time. Similar to our results in HFFs, HCMV infection of SGHPL-4 cells altered the subcellular distribution of β-catenin and caused it to accumulate in a juxtanuclear location.

**Figure 6:**
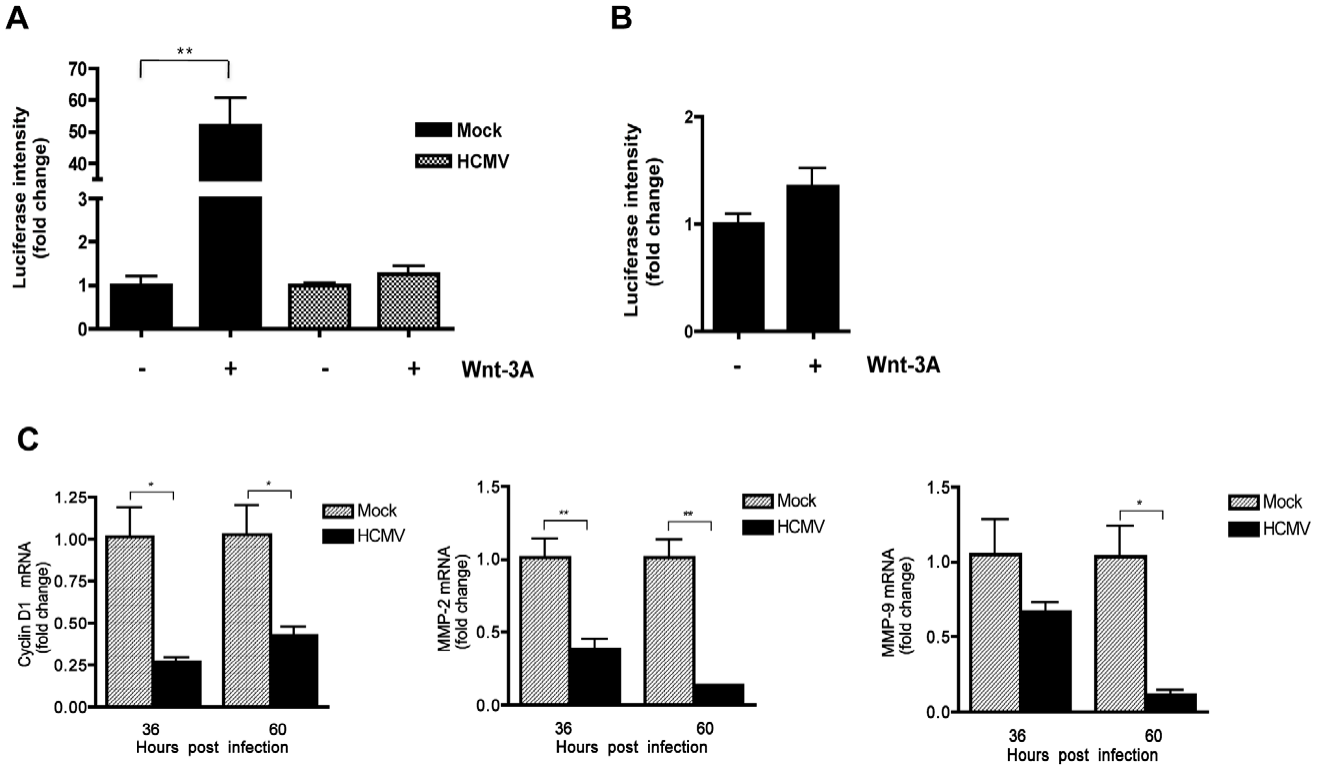
HCMV infection inhibits Wnt/β-catenin transcriptional activity in EVTs in response to Wnt stimulation. (A) SGHPL-4 cells were co-transfected with TOPflash and pRL-TK plasmids and 6 hr later infected with Towne-GFP (MOI of 1–2) or mock-infected. 48 hr after infection, the cells were stimulated with either 150 ng/mL Wnt-3A or PBS control for an additional 12 hr. Luciferase expression in cell lysates was measured and normalized to *Renilla* luciferase activity. Data are presented as the mean ± SEM of results from three independent experiments each performed with duplicate transfections (n = 6). **p<0.01. (B) FOPflash (C) *Cyclin D1*, *MMP-2* and *MMP-9* mRNA levels in SGHPL-4 cells 36 and 60 hr after infection with HCMV-TR (MOI of 1–2) or mock-infection were analyzed by qRT-PCR in triplicate. mRNA levels were normalized to GAPDH expression. The data are presented as fold change (mean +/− SEM) relative to the mock-infected sample at each timepoint. *p<0.05; **p<0.01.

To investigate whether HCMV infection of SGHPL-4 cells also affected the transcriptional activity of β-catenin, cells were transfected with TOPflash or FOPflash and 6 hr later infected with HCMV or mock-infected. Forty-eight hr later, cells were stimulated with Wnt-3A ligand for 12 hr and then analyzed for luciferase expression. Wnt-3A stimulation of uninfected SGHPL-4 cells resulted in ∼50 fold increase in TOPflash activity compared to PBS-treated (Figure 6A), whereas FOPflash activity remained unchanged (Figure 6B). Similar to our results in HFFs, HCMV-infected SGHPL-4 cells showed no response to Wnt-3A (Figure 6A). Consistent with these results, HCMV infection led to modest, but significant downregulation of β-catenin regulated gene expression, including *cyclin D1*, *MMP-2* and *MMP-9*, compared to mock-infected cells (Figure 6C).

**Figure 7:**
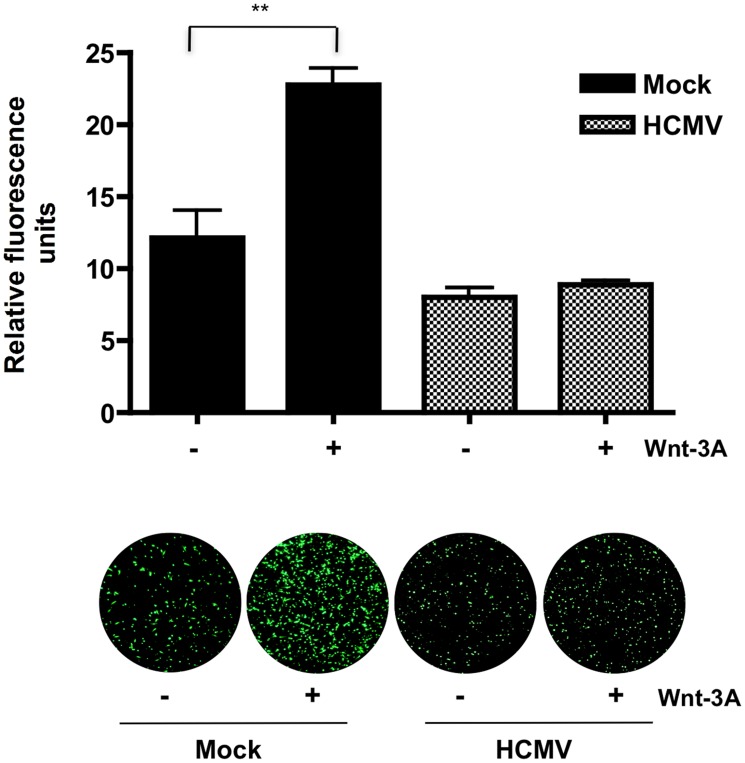
HCMV inhibits Wnt-3A-induced migration of SGHPL-4 EVTs. Migration was assayed using BD FluoroBlok transwell inserts. SGHPL-4 cells were infected with Towne-GFP (MOI of 1–2) or mock-infected for 48 hr prior to the assay. Equal numbers of cells were loaded into the inserts and 150 ng/ml of Wnt-3A or PBS were added to both the upper and lower chambers of the transwell system as indicated. Each condition was performed in triplicate. Migrated wells were stained with calcein AM and visualized with a fluorescent microscope. Average fluorescence intensity was determined by capturing three random fields from each well and measuring their fluorescence intensity using ImageJ software. Data are represented as the mean ± SEM (n = 9). **p<0.001.

To evaluate whether HCMV infection abrogates migration of EVTs upon activation of Wnt/β-catenin signaling, a transwell migration assay was performed (Figure 7). SGHPL-4 cells that had been infected with HCMV and mock-infected controls were allowed to migrate in the presence of 150 ng/ml of Wnt-3A ligand or PBS control for 12 hr. Wnt-3A treatment significantly increased migration of mock-infected controls compared to unstimulated cells. In contrast, HCMV-infected cells were unresponsive to Wnt-3A stimulation and exhibited lower basal levels of migration through the transwell inserts. Together, this suggests that Wnt/β-catenin signaling is also dysregulated in HCMV-infected cytotrophoblasts.

## Discussion

In this study, we report for the first time that HCMV inhibits the canonical Wnt signaling pathway in dermal fibroblasts and human extravillous cytotrophoblasts. We demonstrate that the key player in the pathway, β-catenin, becomes sequestered and degraded in infected cells. Moreover, the transcriptional activity of β-catenin is inhibited in infected cells. Among β-catenin regulated genes are: *cyclin D1* and *c-myc* that are involved in cell cycle regulation and cell proliferation [26], [27]; *DKK-1* that is required for normal embryonic development through its negative feedback regulation of Wnt signaling [30]; as well as *MMP-2*, *MMP-9*, and *MMP-7* that play major roles in cell proliferation, migration, differentiation, angiogenesis, apoptosis and host defense [28], [39].

For β-catenin to drive transcription of target genes it must first translocate to the nucleus [40]. We found that β-catenin protein is prevented from translocating to the nucleus in HCMV-infected cells stimulated with Wnt-3A or LiCl. Instead, β-catenin remains sequestered in a discrete juxtanuclear location. This phenomenon is not cell type specific as we observed similar aggregation of β-catenin in HCMV-infected HFFs (Figure 2A) and SGHPL-4 cells (Figure 5C); nor is it a generalized response to herpesvirus infection, as HFFs infected with herpes simplex virus-1 (HSV-1) failed to cause aggregation of β-catenin (data not shown). The β-catenin aggregates resemble aggresomes that are juxtanuclear depositions of misfolded or damaged proteins that assemble at the MTOC when the cellular proteasome machinery is inhibited or has exceeded its capacity [31]. Accumulation of β-catenin in aggresomes has been demonstrated in hippocampal pyramidal neurons from Alzheimer's patients [41] and in multiple myeloma cells [42]. Ghanevati and Miller suggested that accumulation of β-catenin in aggresomes likely results from proteasomal impairment [41]. However, we found the catalytic activity of the proteasome in HCMV-infected cells to be functional and even increased with progression of infection (Figure 2B). This result is consistent with a previous report [32] and may be explained by the requirement of host proteasome activity for HCMV DNA replication and assembly [43], [44]. Moreover, pharmacological inhibition of the proteasome with MG132 did not lead to perinuclear aggregation of β-catenin (data not shown) suggesting that aggregation of β-catenin in HCMV-infected cells is not a general consequence of proteasomal impairment that hampers normal β-catenin turnover. Further characterization of the β-catenin aggregates by co-immunostaining for specific markers (*e.g.* histone deacetylase 6, γ-tubulin, vimentin) is required to determine if they are indeed aggresomes.

The mechanism(s) by which aggregated β-catenin is eliminated from the infected cells is not clear. Intracellular levels of β-catenin in the cell are maintained through GSK-3β mediated phosphorylation of β-catenin, which targets it for ubiquitination and degradation through the proteasome [36]. However, aggregated proteins are generally poor substrates for the proteasome and are usually eliminated through autophagy [45]. In multiple myeloma cells, β-catenin was found to accumulate in juxtanuclear aggresomes that are cleared by a mechanism involving both autophagy and the ubiquitin proteasome system [42]. We observed a significant decrease in β-catenin protein levels in HFFs infected with HCMV (Figure 3A) however β-catenin protein levels were only slightly decreased in HCMV-infected SGHPL-4 cells (data not shown). These results may indicate that different cell types clear aggregated proteins by different mechanisms or kinetics.

The HCMV viral particle consists of over forty structural and tegument proteins that are delivered to the host cell upon infection and are known to perturb multiple host signaling pathways [46], [47]. HCMV tegument proteins have been shown to induce proteasomal degradation of a number of key cellular proteins to facilitate viral gene expression and modulate cell cycle events [48]–[50]. However, the inability of UV-inactivated HCMV to promote sequestration and degradation of β-catenin (Figure 4) suggest that viral gene expression is required rather than a viral tegument protein or binding of the virion alone. More work is needed to determine which viral gene(s) are responsible for causing this inhibitory effect on Wnt/β-catenin signaling.

The initial events taking place after HCMV infection are presumed to be essential for the establishment of virus replication and viral pathogenesis. HCMV is known to manipulate cell proliferation [10], microtubule networks [51] and proteasome activity [32] to enhance viral replication and assembly. Wnt/β-catenin signaling either regulates or is regulated by these same cellular processes. Tumorigenic gammaherpesviruses, such as Kaposi's sarcoma associated herpesvirus (KSHV) and Epstein-Barr virus (EBV), are well known to activate the canonical Wnt/β-catenin pathway thus promoting cell division and proliferation [52], [53]. HCMV, to our knowledge, is the first human herpesvirus that has been reported to inhibit the Wnt/β-catenin pathway. How dysregulation of Wnt/β-catenin signaling may benefit the virus is unknown, however understanding the effect of the virus on this essential cellular pathway sheds light on HCMV pathogenesis. Wnt signaling is inherently complex. Both the ligands and receptors involved in Wnt signal transduction belong to large multi-gene families, allowing for a large number of possible ligand-receptor interactions that can elicit a variety of intracellular responses (reviewed in [54]). Thus HCMV-mediated dysregulation of canonical Wnt signaling could have different effects depending on the type of cell infected and the cellular context.

Dysregulation of Wnt/β-catenin signaling pathway by HCMV is likely to be of physiological significance particularly during congenital infection. It has been established that intrauterine transmission of HCMV during pregnancy is associated with abnormal placental development caused by impaired differentiation of EVTs. During gestation, these highly specialized cells differentiate into highly proliferative invasive cells that migrate through the decidua and remodel maternal spiral arteries to establish a vascular connection between the mother and the fetus [55]–[57]. HCMV infection of placental EVTs has been proposed to inhibit their ability to differentiate and adequately invade the decidua and could impair their ability to remodel the uterine spiral arteries during pregnancy resulting in shallow placentation [38], [58]–[60]. However, the mechanisms are not well understood.

Prior to differentiating into highly invasive cells, cytotrophoblasts undergo rapid proliferation in order to establish cell-anchoring columns that attach the placenta to the uterine wall. We have previously shown that HCMV markedly inhibits EVT proliferation [38]. HCMV-infected cells arrest in a pseudo G1 phase and fail to enter S phase [11]. High expression of *cyclin D1* is required for initiation of S phase, after which the level of the protein drops. In this study, *cyclin D1* expression that is dependent on β-catenin activation was modestly, but significantly downregulated in HCMV-infected SGHPL-4 cells and HFFs (Figures 6C and 1C, respectively). In agreement with these results, downregulation of cyclin D1 protein levels has been previously reported in HCMV-infected HFFs [10]. Thus downregulation of *cyclin D1* expression as a result of HCMV inhibition of β-catenin transcriptional activity likely contributes to the block in proliferation of infected EVTs.

Invasion of EVTs during placental development involves production of extracellular matrix-degrading metalloproteinases and vasculogenic factors [61], [62]. At the molecular level, these processes are mediated by the canonical Wnt signaling pathway [39], [63]. Studies show that first trimester and term human placental tissues as well as several cytotrophoblast cell lines express high levels of Wnt signaling pathway components including ligands, FZD receptors and transcription factors from the TCF and LRP families [63], [64]. Furthermore, canonical Wnt ligands, including Wnt-3A, have been previously reported to promote motility and invasiveness of the EVT cell line SGHPL-5 and primary EVTs purified from first-trimester placentas, through activation of the Wnt/β-catenin signaling pathway and upregulation of MMP-2 and MMP-9 [19], [62]. In this study we demonstrate that infected SGHPL-4 cells, that are very similar to SGHPL-5 cells [65], fail to migrate in response to Wnt-3A stimulation (Figure 7). In addition, expression of *MMP-2* and *MMP-9* mRNAs was significantly decreased in HCMV-infected SGHPL-4 cells (Figure 6C). Consistent with these results, we previously demonstrated that the secretion and activity of MMP-2 and MMP-9 was dramatically reduced in HCMV-infected SGHPL-4 cells [38]. Therefore, inhibition of Wnt signaling by the virus may be responsible for the observed decreased MMP production and impaired invasiveness in infected EVTs. Our results do not exclude the possibility that additional components of the Wnt/β-catenin pathway or other signaling pathways that influence cytotrophoblast migration and invasion may also be affected by HCMV infection. A recent study by Rauwel *et al.* demonstrates that HCMV inhibits migration and invasion of EVTs through activation of peroxisome proliferator-activated receptor gamma (PPAR-γ), a nuclear receptor that regulates lipogenesis and inflammation [66]. Interestingly, PPAR-γ has been shown to negatively regulate β-catenin [67]. In addition, several growth factors and cell surface proteins have been shown to stimulate cytotrophoblast migration and invasion through activating signaling molecules and pathways such as focal adhesion kinase (FAK), mitogen-activated protein kinase/extracellular signal-regulated kinase (MAPK/ERK), and phosphatidylinositol-3-kinase (PI3K)/AKT (as reviewed in [68]). However, the effect of HCMV infection on the status of these pathways in cytotrophoblasts has not been established.

β-catenin is not only an essential component of canonical Wnt signaling, it is also an integral constituent of adherens junctions where it mediates contact between classical cadherins, α-catenin and the actin cytoskeleton. Our data indicate that membrane-associated β-catenin is also decreased in HCMV infected cells (Figure 3B). Since the E-cadherin/β-catenin complex is very important in maintaining epithelial morphology and integrity [69], its disruption could contribute to the profound changes in cellular morphology observed in HCMV infected cells.

In conclusion, we report for the first time that HCMV infection dysregulates the canonical Wnt/β-catenin signaling pathway in human dermal fibroblasts and placental EVTs. Our study establishes for the first time that HCMV inhibits canonical Wnt signaling by causing sequestration and degradation of endogenous β-catenin, thus preventing its downstream activities. Since the Wnt/β-catenin pathway is an evolutionarily conserved pathway involved in a diverse range of biological functions such as cell cycle control, cell differentiation, embryonic development, placentation and metastasis, the finding that HCMV impairs this pathway becomes globally important for understanding viral pathogenesis, particularly that related to HCMV disease.

## Materials and Methods

### Cell lines, viral strains and infections

Human foreskin fibroblasts (HFFs) were purchased from American Type Culture Collection (ATCC, Manassas, VA) and were cultured in Dulbecco's Modified Eagle Medium (DMEM, Sigma-Aldrich, St. Louis, MO) supplemented with 10% fetal bovine serum (FBS) and 1% penicillin-streptomycin-L-glutamine at 37°C in 5% CO_2_. The human extravillous-like cytotrophoblast cell line SGHPL-4 cells was derived from first trimester chorionic villous tissue and exhibits features of invasive cytotrophoblasts, such as expression of HLA-G, CD9 and cytokeratin-7 [65], [70]. SGHPL-4 cells were maintained in Ham's F10 Nutrient Mix (Invitrogen, Carlsbad, CA) supplemented with 10% FBS (Atlas Biologicals, Fort Collins, CO), 1% penicillin-streptomycin-L-glutamine (Invitrogen) at 37°C in 5% CO_2_. All experiments were carried out using either a laboratory strain of HCMV that expresses GFP from the *UL127* promoter (Towne-GFP), or the HCMV BAC-derived clinical strain TR, both kindly provided by Dr. Dan Streblow (Oregon Health & Science University, OR). All viral strains were propagated in HFFs. For HCMV infections, cells were synchronized by serum starving overnight and infected with HCMV at multiplicity of infection (MOI) of 1 to 2. Briefly, viral inoculum was added to the cells and allowed to adsorb for 90 min at 37°C in 5% CO_2_. The virus inoculum was then removed and replaced with fresh medium containing 0.5% FBS.

### Antibodies and reagents

Primary antibodies: mouse anti-β-catenin (Santa Cruz Biotechnology, Santa Cruz, CA; 1∶500 dilution), mouse anti-β-actin (Abcam, Cambridge, MA; 1∶5,000), mouse anti-CMV IE1/2 (Millipore, Billerica, MA; 1∶200), mouse anti-CMV p65 (Santa Cruz Biotechnology; 1∶500), mouse anti-γ-tubulin (Abcam; 1∶1000), rabbit anti Histone 4 (H4) (Millipore, Billerica, MA; 1∶1000), rabbit anti-GAPDH (Sigma; 1∶5000), rabbit anti-caveolin-1 (Abcam; 1∶500). Secondary antibodies: AlexaFluor antibodies were purchased from Invitrogen and used at 1∶1000 dilution: AlexaFluor 488 goat anti-mouse, AlexaFluor 555 goat anti-mouse. Human recombinant Wnt-3A was purchased from R&D (Minneapolis, MN). Lithium chloride (LiCl) was purchased from Sigma-Aldrich.

### Immunofluorescence

For all immunofluorescence analyses, cells were seeded onto 1.5 mm glass coverslips coated with 0.2% gelatin, and infected with HCMV or mock-infected as described above. At indicated time points after infection, cells were washed with Dulbecco's phosphate-buffered saline (DPBS, Invitrogen) and fixed in 2% paraformaldehyde (Ted Pella, Redding, CA) for 20 min. Cells were washed with DPBS and incubated with 50 mM NH_4_Cl solution in DPBS for 20 min followed by permeablization in 0.1% Triton X-100 for 8 min. Prior to blocking, the cells were incubated with an Fc receptor blocker (Innovex Biosciences, Richmond, CA) for 30 min at room temperature. The cells were then blocked in 5% bovine serum albumin (BSA) for 1 hr at room temperature. After blocking, cells were incubated with primary antibodies diluted in blocking solution at the manufacturer's recommended dilution for 1 hr at room temperature. The cells were washed and incubated for 1 hr with AlexaFluor-conjugated secondary antibodies and 4′-6-Diamidino-2-phenylindole (DAPI) as a nuclear counterstain. Coverslips were washed and mounted with ProLong gold antifade reagent (Invitrogen) and imaged at 40× using a Zeiss Axioplan II microscope (Carl Zeiss, Thornwood, NY, USA). Processing of the acquired images was performed using Adobe Photoshop software.

### Luciferase analyses

To evaluate activation of the Wnt/β-catenin pathway, SGHPL-4 cells were transiently transfected with the TCF/LEF-1 reporter plasmid TOPflash, which contains multimeric TCF/LEF-1 sequences upstream of a firefly luciferase reporter gene. FOPflash plasmid containing mutated TCF/LEF-1 binding sites was used as a specificity control for TOPflash activity. Both TOPflash and FOPflash plasmids were obtained from Millipore (Bedford, MA). A Renilla luciferase-expressing plasmid pRL-TK (Promega, Madison, WI) was co-transfected with both TOPflash and FOPflash to serve as an internal transfection control. For each transfection, SGHPL-4 cells were transfected with 1 µg of either TOPflash or FOPflash DNA and 0.1 µg of Renilla plasmid using the Neon transfection system (Invitrogen) according to the manufacturer's instructions (1 pulse, a pulse width of 20 ms and voltage of 1400 V). Each transfection was performed in triplicate. Six hr after transfection, the cells were infected with HCMV (MOI of 1 to 2) or mock-infected. Forty-eight hr after infection, the cells were stimulated with 150 ng/ml Wnt-3A or PBS vehicle control for an additional 12 hr and cells were harvested. Luciferase activity was assayed with a dual-luciferase reporter assay kit (Promega, Madison, WI) and measured by a Lumat LB 9507 tube luminometer (Berthold Technologies, Bad-Wildbad, Germany). For each sample, the firefly luminescence signal was normalized to the corresponding Renilla signal.

### Migration assay

SGHPL-4 cells were infected with HCMV or mock-infected at an MOI of 1 to 2. Cells were trypsinized 48 hr after infection, collected in serum-free F-10 media and added to the upper side of an 8 µm FluoroBlok 24-well multiwell insert system (BD Discovery Labware, Bedford, MA) at a density of 5×10^4^ cells per insert. Wnt-3A ligand (150 ng/ml) or PBS control was added to both the upper and the lower chambers of the multiwell plate. Each condition was performed in triplicate. The cells were allowed to migrate through the FluoroBlok membrane for 12 hr at 37°C in 5% CO_2_, after which they were fluorescently labeled with calcein AM (Molecular Probes, Eugene, OR) and visualized by fluorescent microscopy. The 12 hr timepoint was empirically determined to be optimal for SGHPL-4 cell migration. Three random fields from each insert were captured with a Nikon TE200 inverted fluorescent microscope (Nikon Instruments, Melville, NY) and the average fluorescence intensity was determined by ImageJ analysis software.

### UV-inactivation of virus

HCMV was UV-inactivated by exposure to 426 mJ of 254 nm UV light in Bio-Rad GS gene linker UV Chamber (Bio-Rad, Hercules, CA). UV inactivation was assured by the absence of HCMV IE1/2 expression in HFFs.

### Quantitative real-time RT-PCR (qRT-PCR)

Total RNA was collected from mock and HCMV-infected cells using the Qiagen RNeasy Kit (Qiagen, Valencia, CA) according to the manufacturer's instructions. RNA (500 ng) was reverse transcribed using Bio-Rad iScript cDNA synthesis kit (Bio-Rad) and PCR reactions were performed using SYBR Green supermix (Bio-Rad) or TaqMan Universal PCR (Applied Biosystems, Carlsbad, CA) master mix and the iCycler Real-Time PCR detection system (Bio-Rad). Oligonucleotide primers (Integrated DNA Technologies, Coraville, IA) used are as follows: cyclin D1 forward (5′-CGCCCTCGGTGTCCTACTTC-3′), cyclin D1 reverse (5′-GACCTCCTCCTCGCACTTCTG-3′); MMP-2 forward (5′-ATGTCGCCCCCAAAACGGACAAAG-3′), MMP-2 reverse (5′-CGCATGGTCTCGATGGTATTCTGG-3′); MMP-9 forward (5′-AGACGGGTATCCCTTCGACG-3′), MMP-9 reverse (5′-AAACCGAGTTGGAACCACGAC-3′); β-catenin forward (5′-T ACAAACTGTTTTGAAAATCCA-3′), β-catenin reverse (5′-CGAGTCATTGCATACTGTCC-3′); DKK1 forward (5′-TTCCAACGCTATCAACCTGC-3′), DKK1 reverse (5′- CAAGGTGGTTCTTCTGGAATACC-3′); c-myc forward (5′- GCCACGTCTCCACACATCAG-3), c-myc reverse (5′-TCTTGGCAGGAGGATAGTCCTT-3′); 36B4 forward (5′-TGGAGACGGATTACACCTTC-3′), 36B4 reverse (5′-CTTCCTTGGCTTCAACCTTAG-3′); Human GAPDH mRNA was measured using a TaqMan Gene Expression Assay according to the manufacturer's protocol (Applied Biosystems; Carlsbad, CA). Prior to performing real-time PCR on experimental samples, primer concentrations were optimized to provide equal priming efficiency (∼100%) for each primer pair. Negative controls, including cDNA reactions without reverse transcriptase or RNA and PCR mixtures lacking cDNA were included in each PCR. Following amplification, specificity of the reaction was confirmed by melt curve analysis. Relative quantitation was determined using the comparative C_T_ method with data normalized to GAPDH or 36B4 and calibrated to the average ΔC_T_ of mock-infected control at the specified time point.

### Western blotting

For Western blot analyses, cells were lysed in SDS lysis buffer (62.5 mM Tris-HCl, 2% SDS, 10% glycerol, 50 mM DTT) supplemented with protease inhibitor cocktail (Roche Chemicals, Indianapolis, IN). The lysates were sonicated briefly and protein content was determined by Bradford assay (Bio-Rad). Equivalent amounts of protein were separated by SDS-PAGE and transferred to nitrocellulose. The blots were blocked with 5% BSA and incubated overnight with primary antibodies diluted in blocking solution at the recommended dilutions at 4°C. Following three washes with Tris-buffered saline (TBS), blots were incubated with horseradish peroxidase-conjugated anti-mouse or anti-rabbit antibodies (Invitrogen) diluted in 5% BSA at 1∶10,000. After washing in TBS, proteins were detected using SuperSignal chemiluminescent substrate (Pierce Biotechnology, Rockford, IL) according to the manufacturer's instructions. For densitometric analysis of Western blot images, density profiles of the bands were measured using ImageJ software.

### Cell fractionation

Membrane/cytoskeletal, cytoplasmic and nuclear fractions were isolated from HFFs that had been mock and HCMV-infected for 48 hr, using the Qproteome cell compartment kit (Qiagen) according to the manufacturer's protocol. Resulting fractions were separated on SDS-PAGE and immunoblotted for β-catenin. Histone H4, GAPDH and caveolin-1 were used to assess the purity of the cell fractions as previously described [33]–[35].

### Proteasome activity assay

HFF cells (1.5×10^4^) were plated in a 96-well tissue culture plate in 100 ìl DMEM supplemented with 10% FBS. The cells were infected with HCMV-TR (MOI of 1 to 2) or mock-infected. Cells were analyzed at 24, 48 and 72 hr after infection using a Proteasome Assay kit (Cayman Chemical, Ann Arbor, MI) that measures the catalytic activity of the 26S proteasome, according to the manufacturer's instructions. The fluorescence intensity was measured at an excitation of 360 nm and emission of 480 nm using a fluorescence plate reader.

### Statistical analysis

Data from HCMV-infected groups were compared to mock-infected groups and significant differences were determined by Student's t-test or one-way analysis of variance (ANOVA) followed by Tukey's post hoc *t* test using GraphPad Prism 4 software. Data are presented as the means ± standard error of the means (SEM).
